# Prognostic role of tumour volume and downstaging response on outcome after liver transplantation for colorectal liver metastases: retrospective study

**DOI:** 10.1093/bjsopen/zraf170

**Published:** 2026-01-21

**Authors:** Håvard Bjørke Jenssen, Svein Dueland, Tor Magnus Smedman, Harald Grut, Andreas Abildgaard, Pål D Line, Trygve Syversveen

**Affiliations:** Division of Radiology and Nuclear Medicine, Oslo University Hospital, Oslo, Norway; Transplant Oncology Research Group, Division of Surgery and Specialized Medicine, Oslo University Hospital, Oslo, Norway; Transplant Oncology Research Group, Division of Surgery and Specialized Medicine, Oslo University Hospital, Oslo, Norway; Department of Oncology, Oslo University Hospital, Oslo, Norway; Transplant Oncology Research Group, Division of Surgery and Specialized Medicine, Oslo University Hospital, Oslo, Norway; Department of Radiology, Vestre Viken Hospital Trust, Drammen, Norway; Division of Radiology and Nuclear Medicine, Oslo University Hospital, Oslo, Norway; Transplant Oncology Research Group, Division of Surgery and Specialized Medicine, Oslo University Hospital, Oslo, Norway; Section for Transplant Surgery, Department of Transplantation Medicine, Oslo University Hospital, Oslo, Norway; Institute of Clinical Medicine, University of Oslo, Oslo, Norway; Division of Radiology and Nuclear Medicine, Oslo University Hospital, Oslo, Norway

**Keywords:** survival predictors, radiological response, hepatic recurrence, transplant eligibility, multimodal treatment, overall survival

## Abstract

**Background:**

The incidence of colorectal cancer is increasing, and the liver remains the predominant site for metastases. Whereas liver resection is the standard treatment for colorectal liver metastases (CRLMs), liver transplantation (LT) has re-emerged as a viable option for selected patients. The aim of this study was to investigate whether tumour volume and changes in tumour volume during chemotherapy before transplantation predict overall survival.

**Methods:**

Patients who underwent LT for CRLMs between November 2006 and August 2020 were included. Tumour volumes were measured via manual segmentation on computerized tomography scans at baseline, at maximum tumour volume, and immediately before LT. Response to chemotherapy was assessed using Response Evaluation Criteria in Solid Tumours (RECIST) criteria, and the heterogeneous response was noted to investigate whether this subgroup performs differently. Receiver operating characteristic analysis was conducted to determine a tumour volume cut-off value for predicting overall survival. Overall survival between groups was compared using Kaplan–Meier curves and log rank test.

**Results:**

Fifty-nine patients who underwent LT for CRLMs were analysed retrospectively. Receiver operating characteristic analysis revealed that final tumour volume at time of LT was a strong predictor of 5-year overall survival (area under the curve= 0.789), with a 35 mL cut-off providing optimal clinical discrimination. Patients achieving a final tumour volume below 35 mL, either consistently or via downstaging, demonstrated significantly improved survival compared with those with persistently high tumour volumes (4.54 years *versus* 2.17 years; *P* < 0.001). Heterogeneous responses to chemotherapy were associated with poorer prognosis with no patients surviving beyond 2.16 years (*P* < 0.001).

**Conclusion:**

Dynamic tumour assessment, particularly measuring tumour volume to below 35 mL, is an important prognostic marker in LT for CRLMs.

## Teaser

This study evaluates the prognostic role of tumour volume and response to downstaging in 59 patients undergoing liver transplantation for colorectal liver metastases. A tumour volume threshold of 35 mL before transplantation was associated with improved 5-year survival, and patients who reached this threshold through successful downstaging had outcomes comparable to those with consistently low tumour burden. Heterogeneous lesion response to chemotherapy identified patients at higher risk, highlighting the value of volumetric and dynamic tumour assessment in patient selection.

## Introduction

Colorectal cancer is on the rise in the Western world, with an annual increase of 2% among individuals under 50 years old in the USA^1^. The liver is the primary site for colorectal cancer metastases. Liver resection is the only widely accepted curative treatment for colorectal liver metastases (CRLMs)^2^. Liver transplantation (LT) for technically unresectable CRLMs was initially explored in the 1990s but was abandoned due to poor long-term survival outcomes^3^. Since 2006, LT for unresectable CRLMs has been revisited by this study’s research group in the SECA studies^4^. These studies suggest that survival rates comparable to those seen with conventional indications can be achieved in selected patients with liver-only CRLMs^5^.

The success of LT in hepatocellular carcinoma (HCC), particularly within the Milan criteria, has provided a conceptual framework for patient selection in CRLMs. In HCC, expanding access to LT through downstaging strategies has become increasingly accepted, and the concept of an upper tumour burden threshold has gained traction^6^. Downstaging patients with HCC beyond the Milan criteria—primarily through locoregional treatments—is increasingly used to expand access to LT. Studies^7,8^ have also explored whether there is an upper limit of tumour burden beyond which successful LT becomes unrealistic. These principles are now being explored in the CRLMs setting as well. A recent randomized trial^9^ has provided level 1 evidence of the survival benefit of LT over chemotherapy for unresectable CRLMs, with a 5-year overall survival of 73.3% in the LT group compared with 9.3% in patients treated with chemotherapy alone. In liver resection studies^10^, heterogeneous response to neoadjuvant therapy has been associated with poor prognosis. As LT moves closer to standard of care for selected CRLMs patients, refining selection criteria to maximize graft utility and avoid futile transplantation becomes essential.

This study investigated the prognostic impact of tumour volume and response to systemic therapy before LT for CRLMs. With the advent of volumetric assessment of radiological images, this study investigated whether total tumour volume, measured on a final computerized tomography (CT) scan before transplantation, and changes in volume during downstaging are predictive of survival. This study also explored whether heterogeneous response to chemotherapy is associated with outcomes following LT. As previous studies have established a metabolic tumour volume cut-off of 70 mL as prognostic, this study examined whether a similar threshold can be defined using morphologic tumour volume to inform future patient selection.

## Methods

### Patient selection

The main inclusion criteria were confirmed CRLMs that were deemed technically unresectable, good performance status (ECOG score of 0 or 1), previous chemotherapy for downstaging, and completion of primary tumour resection. Patients who underwent LT for CRLMs between November 2006 and August 2020 were included in the study.

The SECA and RAPID trials were approved by the Regional Ethics Committee and registered at ClinicalTrials.gov (registration numbers NCT01311453 for SECA-I, NCT01479608 for SECA-II, and NCT02215889 for RAPID). All patients provided written informed consent.

### Image selection, segmentation, and tumour volume measurement

Contrast-enhanced CT images of the liver obtained in the portal venous contrast medium phase at baseline (time of diagnosis), at the point of maximum tumour volume, and the final imaging study before transplantation were assessed. The CT scan at the time of maximum tumour volume was manually identified by one radiologist (TS) comparing images from all CT examinations performed between baseline and transplantation. In cases of uncertainty, tumour volume was calculated across multiple CT scans to identify the time of maximum tumour volume.

**Fig. 1:**
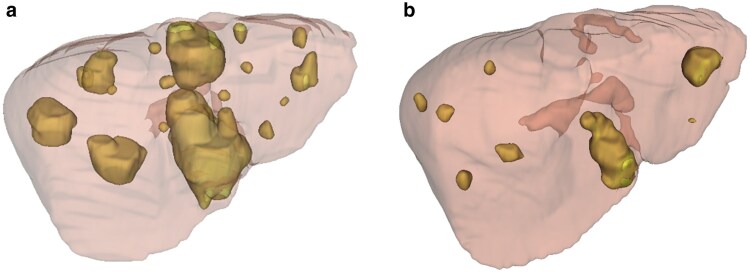
Segmentation of a liver with tumours highlighted in yellow The liver parenchyma is semi-transparent, rendered in light brown. (**a**) Patient at maximum tumour volume, and (**b**) same patient after chemotherapy downstaging, with a reduction in tumour size.

The images were manually segmented for tumour. An experienced abdominal radiologist (HBJ) performed all segmentations. Volumetric measurements were derived from the segmentation masks. An example of tumour segmentation is given in *Fig. 1*.

Patients were divided into three groups: those whose largest single tumour diameter was always below 5.5 cm, those whose tumour diameter was above 5.5 cm at maximum but decreased to below 5.5 cm before LT, and those whose largest diameter remained above 5.5 cm throughout.

Cystic and calcified components of the metastases were included in the volume measurements. For focal lesions where the diagnosis of metastasis was uncertain, corresponding magnetic resonance imaging was used for clarification.

### RECIST criteria and heterogeneous response

Response Evaluation Criteria in Solid Tumours (RECIST) evaluations were performed by a single radiologist (TS) in accordance with the revised RECIST guidelines (version 1.1)^11^, with a minor modification: target lesions were not redefined if chemotherapy regimens changed. Two target lesions were identified on the baseline examination and monitored throughout the study, regardless of chemotherapy line changes, until the time of LT.

In cases where target lesions disappeared from baseline to the time of maximum tumour volume due to resection or ablation, new target lesions were selected at the time of maximum volume. The final response to treatment according to RECIST was estimated by scoring the second last and last examination before LT. Patients were scored as having stable disease (SD), partial response (PR) and progressive disease (PD), and the overall survival (OS) was compared between the groups. All patients were also assessed for the presence of heterogeneous response to chemotherapy, as described by Brunsell^10^, using a threshold of 3 mm diameter change for lesion growth or shrinkage.

### Statistical analysis

Statistical analyses were performed using IBM^®^ SPSS^®^ Statistics for Windows, version 29.0.2.0 (Armonk, NY, USA: IBM Corp). Receiver operating characteristic (ROC) analysis was conducted to determine a tumour volume cut-off value for predicting OS. OS was defined, as in previous studies, as the time from transplantation to either patient death or the end of follow-up (5 years). OS between groups was compared by using Kaplan–Meier curves and log rank test. *P* < 0.05 was considered significant.

## Results

### Patient characteristics

Patient data for this study were derived from the SECA-I and SECA-II trials, with two additional patients from the RAPID study, with a potential total of 60 patients. A total of 59 patients were included after image review, as one patient was excluded due to unavailable radiological imaging. One patient from the SECA 2A-arm deemed resectable was randomized to transplantation; the remaining 58 were deemed unresectable at the time of transplantation (*Table 1*). Further details have been published in the Dueland *et al*. study^12^.

**Table 1:** Patient characteristics with patient age, sex ratio and average tumour volumes

Age at LT	57 (30–71)
Sex ratio (male:female)	34:25 (57%:43%)
Tumour volume at baseline (ml)	52.64 (0.10–1552.20)
Tumour volume at max (ml)	149.70 (1.84–2250.90)
Tumour volume at last CT before LT (ml)	13.89 (0.13–2250.90)

Values are median (range) unless otherwise stated. LT, liver transplantation.

Ten of 59 patients underwent liver resections and/or ablations before transplantation with curative intent. With subsequent progression, these patients became unresectable. Two of these patients had resections between the maximum tumour volume CT time point and their LT date, and thus had resection that in retrospect could be considered part of their ‘downstaging regime’.

Some patients had imaging studies that represented more than one category; for example, the CT with maximum tumour volume was also the last CT before LT. This resulted in fewer than 177 CT scans being analysed. Ultimately, 147 unique CT scans were assessed.

### Survival analysis: RECIST *versus* tumour volume assessment

ROC analysis was performed on tumour volumes at baseline, at the time of maximum tumour volume, and at the last examination before transplantation to evaluate their predictive value for OS. The analysis showed that the final pretransplant tumour volume had higher predictive accuracy for OS than the maximum tumour volume.

The percentage reduction in tumour volume from maximum to transplantation was not prognostic for survival, with an area under the curve (AUC) of 0.337.

The tumour volume measured at the last examination before transplantation yielded an AUC of 0.762 for predicting 5-year OS (*Fig. S1*). Youden’s J analysis identified an optimal volumetric cut-off of 15 mL (J = 2.535). The J-index remained relatively stable between 10 mL (J = 2.512) and 36 mL (J = 2.508); therefore, a cut-off of 35 mL was selected for further analyses to stratify patients by predicting the 5-year OS while maintaining clinical relevance.

**Fig. 2:**
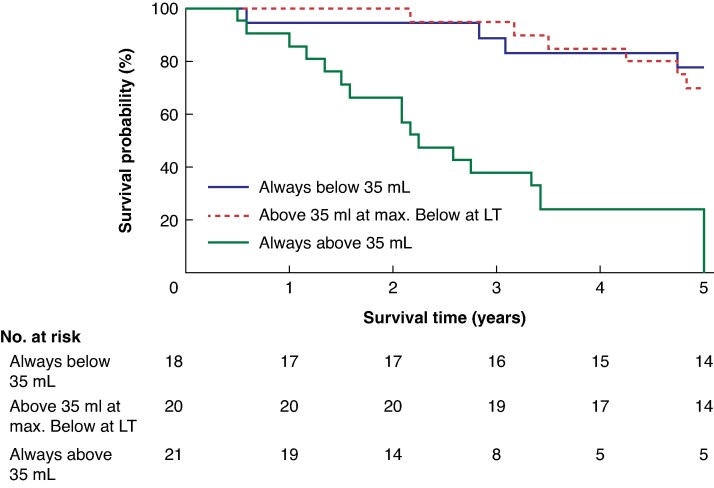
Kaplan–Meier survival curves comparing survival based on the pattern of tumour volume changes before liver transplantation Groups include patients with tumour volume always below 35 ml (blue), those whose tumour volume was above 35 ml at maximum but below 35 ml at liver transplantation (red), and those with tumour volume consistently above 35 ml (green). (*P* < 0.0001). LT, liver transplantation.

Patients were subsequently divided into three groups: tumour volume consistently < 35 mL, tumour volume > 35 mL at maximum but <35 mL before transplantation, and tumour volume consistently > 35 mL. Kaplan–Meier analysis demonstrated significantly shorter OS for group 3 compared with groups 1 and 2 (*P* < 0.001; *Fig. 2*).

### Tumour size and RECIST evaluation: SD, PR, or PD

A similar analysis was performed using tumour diameter, applying a 5.5 cm cut-off previously used in comparable studies. Patients were divided into three groups: largest tumour diameter consistently < 5.5 cm, tumour diameter > 5.5 cm at maximum but < 5.5 cm before liver transplantation, and tumour diameter consistently > 5.5 cm.

Kaplan–Meier analysis showed no statistically significant difference in OS between the three groups (*P* = 0.436; *Fig. S2*). Eleven patients had tumour diameters consistently > 5.5 cm.

**Fig. 3:**
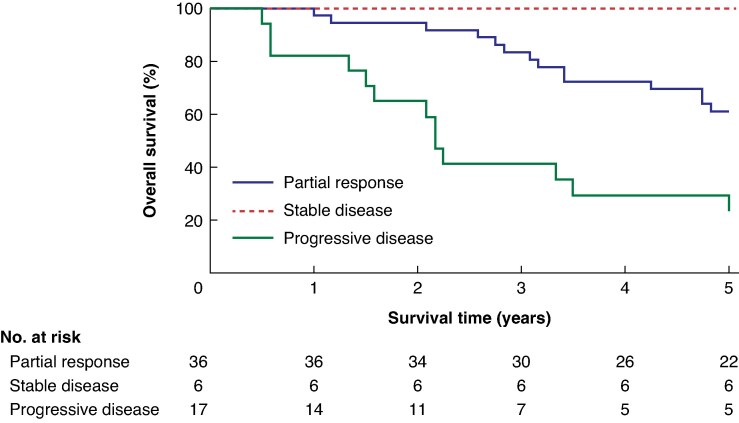
Kaplan-Meier survival curves comparing survival based on RECIST (Response Evaluation Criteria in Solid Tumours) evaluation at the last examination before liver transplantation Groups include partial response (blue), stable disease (red), and progressive disease (green). (*P* < 0.0001).

RECIST 1.1 response categories at the last pretransplant imaging were classified as PR, SD, or PD. Median OS was 60 months for both PR and SD groups and 26.5 months for the PD group (*P* < 0.001; *Fig. 3*).

**Fig. 4:**
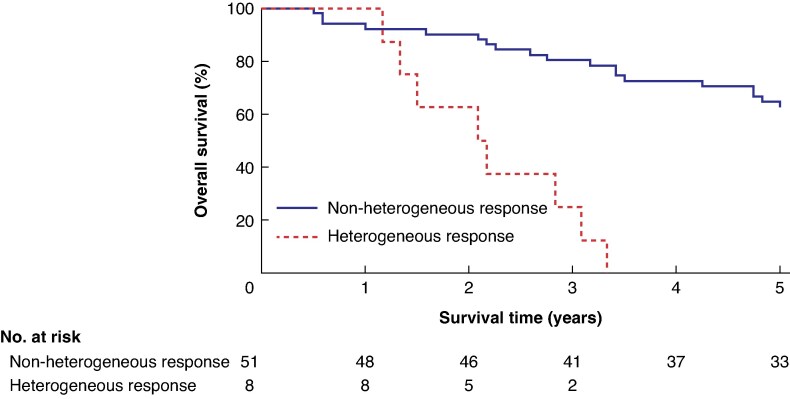
Kaplan-Meier survival curves comparing survival based on heterogeneous status before liver transplantation Groups include non-heterogeneous response (red) and heterogeneous response (blue), with the latter group displaying censored data points. (*P* < 0.0001).

Eight patients demonstrated a heterogeneous response between the time of maximum tumour volume and the final pretransplant examination. Six of these patients also met criteria for progression according to RECIST 1.1. A heterogeneous response was associated with reduced OS (*P* < 0.001; *Fig. 4*), although the subgroup size was limited (8), and only two patients exhibited a heterogeneous response without concurrent PD.

## Discussion

This study highlighted the prognostic relevance of dynamic tumour assessment during the oncological treatment phase in patients with unresectable CRLMs evaluated for LT. The tumour volume measured immediately before transplantation was an important predictor of OS beyond 5 years after LT, demonstrating an acceptable AUC of 0.789 for the cut-off value of 35 mL. This threshold effectively stratified patients into more distinct prognostic groups while maximizing patient eligibility for transplantation without compromising the statistical robustness of selection. Regarding tumour size and in contrast to previous studies that applied a dichotomized cut-off at 5.5 cm and also had a different time cut-off, this study employed a three-group model for tumour size, which may explain the lack of a significant association with survival in the main analysis.

Downstaging using systemic chemotherapy and locoregional interventions to reduce high tumour volumes plays a crucial role in patient outcomes^13^. Successful downstaging was associated with improved OS, comparable to patients whose tumour volumes were always below 35 mL or obtained this volume due to downstaging (*Fig. 2*). This suggests that neoadjuvant treatments can effectively modify disease course and that reducing tumour volume below a critical threshold may serve as a surrogate marker for improved prognosis. For future studies one might also consider looking at patient groups that have undergone intra-arterial treatment options like transcatheter arterial chemoembolization, transarterial radio embolization, and PUMP, as none of this study’s patients had these performed before LT.

Alongside volumetric analysis, evaluating the chemotherapy response offered additional insight into patients with heterogeneous outcomes, where some lesions shrank or disappeared whereas others progressed. Although the number of patients with such heterogeneous responses was limited, the association with poor survival outcomes is notable, with no patients surviving 5 years and in line with similar studies in liver resection for CRLMs, suggesting that a non-uniform response to therapy may be indicative of an aggressive tumour phenotype or subclones of non-responsive tumour cell populations^14^. There was a major difference in predictive efficacy of volumetry *versus* standardized diameter measurements with a non-significant *P*-value associated with the diameter measurement method. In the future, as volumetry becomes more widely used in daily practice due to the introduction of artificial intelligence-based segmentation solutions, it may replace traditional measurements performed today. Consequently, the combination of quantitative volumetric data with qualitative assessments of treatment response adds an important layer of nuance to pretransplant evaluations.

The integration of final tumour volume measurements with an assessment of response heterogeneity offers a more comprehensive and individualized approach to patient evaluation. Such an approach could lead to more consistent and objective selection criteria across transplant centres, thereby improving patient selection. Moreover, by identifying a volumetric threshold, this study provides a starting point to establish a metric that could possibly be incorporated into existing clinical protocols, helping to guide treatment decisions in a more standardized manner.

It is important to acknowledge several limitations of this study. The retrospective design and the relatively small sample size may introduce selection biases that could affect the generalizability of the results. Another limitation was the absence of remnant liver volume data. Furthermore, the impact of downstaging by resection or ablation has not been well studied previously, nor was it in this study. Furthermore, as the study was retrospective, there was no standardization of CT time points, making comparisons less reliable, and this study lacked external validation.

In conclusion, this study identified final tumour volume as a key predictor of survival following LT for unresectable CRLMs, with 35 mL emerging as a clinically meaningful threshold. Successful downstaging below this volume conferred significant survival benefits, whereas heterogeneous treatment responses signalled increased risk of recurrence and mortality. These results argue for a multifaceted evaluation approach that combines quantitative volumetry with qualitative response assessments to optimize transplant selection and outcomes.

## Supplementary Material

zraf170_Supplementary_Data

## Data Availability

Upon request to corresponding author.
