# Supplementary material for: Prognostic role of tumour volume and downstaging response on outcome after liver transplantation for colorectal liver metastases: retrospective study
Source: BJS Open. 2026 Jan 21;10(1):zraf170. doi: 10.1093/bjsopen/zraf170 (PMC12822776; doi:10.1093/bjsopen/zraf170)
Supplement: zraf170_Supplementary_Data [file zraf170_supplementary_data.zip › Supplementary-Material_revision.docx]

**Title: The Prognostic Role of Tumour Volume and Downstaging Response on Outcome after Liver Transplantation for Colorectal Liver Metastases.

Authors:**Håvard Bjørke Jenssen (1)
Svein Dueland (2)
Tor M Smedman (2,3)
Harald Grut (2,4)
Andreas Abildgaard (1)
Pål D Line (2,5,6)
Trygve Syversveen (1)

**Affiliated departments and institutions:**
1. Division of Radiology and Nuclear Medicine, Oslo University Hospital, Oslo, Norway
2. Transplant Oncology Research Group, Division of Surgery and Specialized Medicine, Oslo University Hospital, Oslo, Norway
3. Department of Oncology, Oslo University Hospital, Oslo, Norway
4. Department of Radiology, Vestre Viken Hospital Trust, Drammen, Norway
5. Section for Transplant Surgery, Department of Transplantation Medicine, Oslo University Hospital, Oslo, Norway
6. Institute of Clinical Medicine, University of Oslo, Oslo, Norway

**Corresponding author**: Håvard Bjørke Jenssen. Address: Rikshospitalet, Sognvannsveien 20, 0372, Oslo, Norway. Email: [haajen@ous-hf.no](mailto:haajen@ous-hf.no)

**Supplementary Materials - Index**

| **Supplementary Figures and Tables** |  |
| --- | --- |
| Supplementary Figure 1 | *page 3* |
| Supplementary Figure 2 | *page 3* |
|  |  |

**Supplementary Figures and Tables**

**Supplementary Figure 1.** Kaplan-Meier survival curves comparing survival based on the pattern of tumour size changes prior to liver transplantation. The groups include: patients with tumour size always below 5.5 cm (blue), those who started above 5.5 cm but decreased to below 5.5 cm at transplantation (red), and those with tumour size consistently above 5.5 cm (green). (p-value 0,436)


**Supplementary Figure 2.** Receiver operating characteristic curve demonstrating the predictive value of final tumour volume prior to transplantation for 5-year overall survival (AUC 0.762). The sensitivity and specificity values are plotted to assess the diagnostic accuracy of tumour volume in predicting post-transplant survival outcomes.

**References**
