# Supplementary figures and images for: Prognostic role of tumour volume and downstaging response on outcome after liver transplantation for colorectal liver metastases: retrospective study
Source: BJS Open. 2026 Jan 21;10(1):zraf170. doi: 10.1093/bjsopen/zraf170 (PMC12822776; doi:10.1093/bjsopen/zraf170)

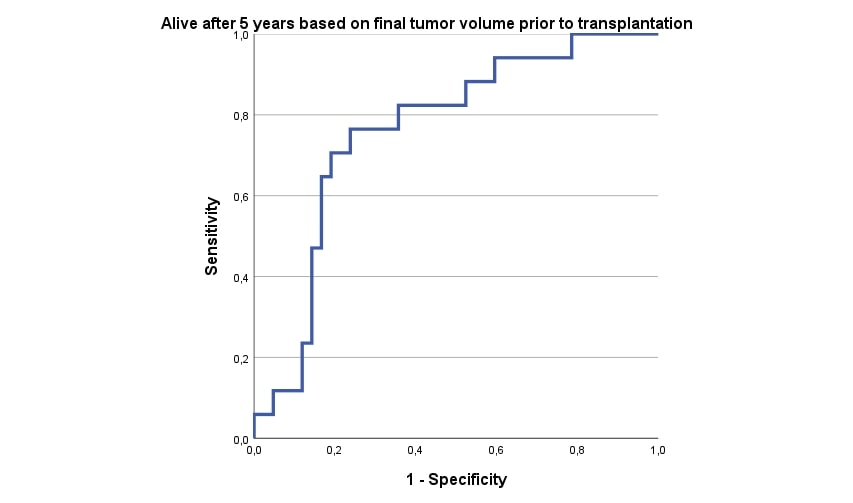

Supplement: zraf170_Supplementary_Data [file zraf170_supplementary_data.zip › Supplementary figure 1.jpg]

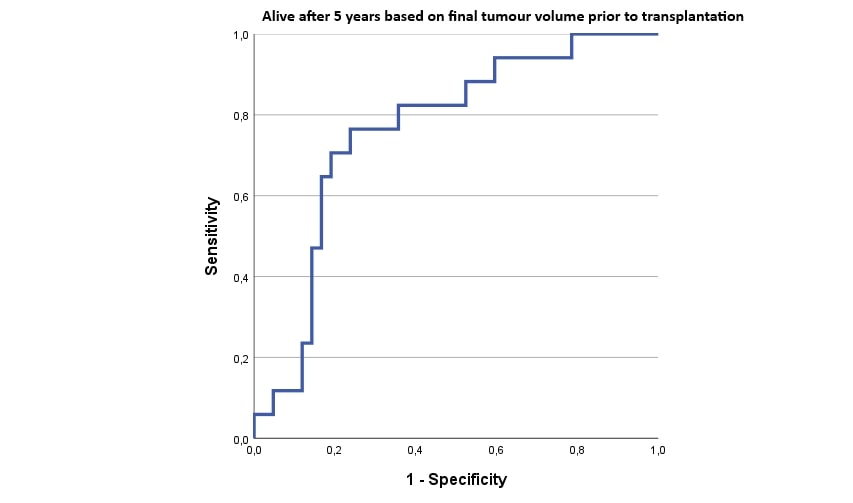

Supplement: zraf170_Supplementary_Data [file zraf170_supplementary_data.zip › Supplementary_Figure 2_reviewed.jpg]
